# Evaluation of normalization methods for cDNA microarray data by *k*-NN classification

**DOI:** 10.1186/1471-2105-6-191

**Published:** 2005-07-26

**Authors:** Wei Wu, Eric P Xing, Connie Myers, I Saira Mian, Mina J Bissell

**Affiliations:** 1 Life Sciences Division, Lawrence Berkeley National Laboratory, Berkeley, CA 94720, USA; 2 Dorothy P. and Richard P. Simmons Center for Interstitial Lung Disease, Division of Pulmonary, Allergy and Critical Care Medicine, University of Pittsburgh Medical Center, Pittsburgh, PA 15213, USA; 3 Center for Automated Learning and Discovery and Language Technology Institute, School of Computer Science, Carnegie Mellon University, Pittsburgh, PA 15213, USA

## Abstract

**Background:**

Non-biological factors give rise to unwanted variations in cDNA microarray data. There are many normalization methods designed to remove such variations. However, to date there have been few published systematic evaluations of these techniques for removing variations arising from dye biases in the context of downstream, higher-order analytical tasks such as classification.

**Results:**

Ten location normalization methods that adjust spatial- and/or intensity-dependent dye biases, and three scale methods that adjust scale differences were applied, individually and in combination, to five distinct, published, cancer biology-related cDNA microarray data sets. Leave-one-out cross-validation (*LOOCV*) classification error was employed as the quantitative end-point for assessing the effectiveness of a normalization method. In particular, a known classifier, *k*-nearest neighbor (*k*-NN), was estimated from data normalized using a given technique, and the *LOOCV *error rate of the ensuing model was computed. We found that *k*-NN classifiers are sensitive to dye biases in the data. Using **N**ONRM and GMEDIAN as baseline methods, our results show that single-bias-removal techniques which remove either spatial-dependent dye bias (referred later as spatial effect) or intensity-dependent dye bias (referred later as intensity effect) moderately reduce *LOOCV *classification errors; whereas double-bias-removal techniques which remove both spatial- and intensity effect reduce *LOOCV *classification errors even further. Of the 41 different strategies examined, three two-step processes, I**G**LOESS-S**L**FILTER**W7**, I**ST**SPLINE-S**L**LOESS and I**G**LOESS-S**L**LOESS, all of which removed intensity effect globally and spatial effect locally, appear to reduce *LOOCV *classification errors most consistently and effectively across all data sets. We also found that the investigated scale normalization methods do not reduce *LOOCV *classification error.

**Conclusion:**

Using *LOOCV *error of *k*-NNs as the evaluation criterion, three double-bias-removal normalization strategies, I**G**LOESS-S**L**FILTER**W7**, I**ST**SPLINE-S**L**LOESS and I**G**LOESS-S**L**LOESS, outperform other strategies for removing spatial effect, intensity effect and scale differences from cDNA microarray data. The apparent sensitivity of *k*-NN *LOOCV *classification error to dye biases suggests that this criterion provides an informative measure for evaluating normalization methods. All the computational tools used in this study were implemented using the R language for statistical computing and graphics.

## Background

Molecular profiling technology allows for the simultaneous assaying of the abundance of tens of thousands of transcripts in a biological sample. Once these abundance values have been obtained for many samples, prevalent higher-order data analyses may include clustering, classification, feature selection, and network estimation. A variety of algorithms seeking to address these higher-order tasks have been investigated and applied, to interpret gene expression patterns and to generate biological predictions. However, the accuracy of these predictions may depend on the low-level transformations utilized to produce abundance values from raw measurements, *i.e.*, data pre-processing may be a critical factor in determining the validity and success of downstream studies. Some key pre-processing steps for profiling data include image quantification and normalization. Several image analysis software (e.g., GenePix and SPOT) have been designed for image analysis of the spots on microarrays [1,2]. Background estimation has also been considered as an important issue in image quantification, however, evidence [2,3] showed that 'inappropriate' local background adjustment could add noise into the microarray data and thus be detrimental to the downstream studies. Background adjustment, therefore, is still an issue to be resolved. After image analysis, normalization usually needs to be performed. It is a procedure designed to minimize the unwanted variations in measurements arising from the technology, but to retain the intrinsic biological variations, and is also the focus of this work. In this study, we examined normalization in the context of a particular transcriptional profiling platform, cDNA microarrays [4-6], and the specific analytical task of classifying biological samples characterized by gene expression profiles.

**Figure 1:**
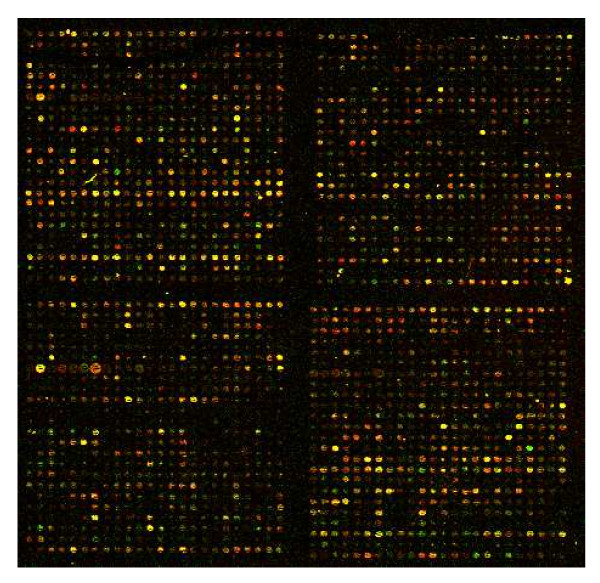
**A scanned image of an illustrative cDNA microarray**. The configuration (layout of spots) can be described via a previously defined notation encompassing four numbers (ngr, ngc, nsr, nsc) [12]. A print-tip (PT) group is a set of spots arranged in a grid with "nsr" rows and "nsc" columns. A microarray is a set of PT groups arranged in a pattern of "ngr" rows and "ngc" columns. The configuration of the microarray shown is (ngr = 2, ngc = 2, nsr = 24, nsc = 24), *i.e.*, 2 × 2 PT groups each composed of 24 × 24 spots. The terms "local" and "global" level refer to the spots in a PT group and the entire microarray respectively.

In cDNA microarray-based investigations, RNA from two samples are reverse-transcribed and labeled with distinct (red and green) fluorescent dyes, then hybridized to a microarray spotted with DNA sequences ("probes"). An ensuing scanned image of the microarray is processed to yield an intensity measurement for each dye at every spot (Figure 1). If *R *and *G *are the spot-specific, quantitated, fluorescent intensities of the target and reference expression signals respectively, relative gene expression is defined as the log ratio *M *= log_2_(*R */ *G*), and average expression is the log intensity . Based on different biological assumptions and design principles, many normalization methods for cDNA microarray data have been proposed. Global normalization techniques adjust the center (*e.g.*, mean or median) of the distribution of the log ratio *M *values on each microarray to a constant [1,7-9]. These methods, however, do not correct for any intensity- or spatial effect.

A variety of techniques have been proposed to remove intensity effect. A non-linear approach employs robust locally weighted regression (**lowess**) [10] to smooth the dependence of log ratios on intensities [4,11,12]. The basic assumption of this approach is either that the majority of genes are not differentially expressed, or that genes are influenced by random effects (*i.e.*, the numbers of up-regulated and down-regulated genes are similar) [4,11,12]. A '**qspline**' method uses a target array to adjust *R *and *G *values so that their distribution is similar for all arrays [13], but the performance of this method may depend upon the choice of the baseline array [14]. A composite method employs both external control samples and total genes on a microarray to remove intensity effect [15]. To relax critical biological assumptions, 'housekeeping-gene'-related methods first identify non-differentially-expressed genes, and then use these genes for normalization [16-18]. Semi-linear models are designed to account for the effects of print-tips (PTs), signal intensity, and differences in gene expression levels jointly in a single model [19,20].

The removal of intensity effect at the PT level can partially remove spatial effect [4,11]. To remove spatial effect more completely, the dependence of *M *values on physical position can be smoothed using **lowess **[12], or can be eliminated using weighted mean [13] or median filter methods [17], both of which assume that differentially expressed genes are not co-localized in the neighboring spots. Since spatial- and intensity effect may be mutually dependent, a method that removes global spatial effect and global intensity effect in a single step has been proposed [21].

Whereas the above location normalization methods remove spatial- and intensity effect, scale normalization methods adjust differences in the scale of *M *values within and/or between microarrays. The assumption is that since the majority of genes are not differentially expressed, the scale of their *M *values should be constant. A robust estimate of the scale factor for scale normalization is median absolute deviation [15].

Normalization approaches seek to ensure that dye effect is removed, while biological variations are retained. Spatial- and intensity effect and scale effect arise from printing, hybridization, scanning, or other technical factors, and can mask the signals arising from genuine biological variations in gene expression. Visual aids used to assess the effectiveness of normalization methods [11,13,15,21] include scatter plots of log ratio (*M*) versus average log intensity (*A*) ("MA plots"). Spatial plots are a color-coded representation of each spot on a microarray that depicts *M *values, or a quality (*e.g.*, shape, size) measure of some test statistic. These two types of diagnostic plots [4,21] suggest that raw *M *values are often biased estimates of relative expression and that the dye intensities per spot need to be adjusted. Quantitative criteria used to assess the robustness of normalization methods in removing dye effect include (i) rank variations of spot intensity in non-normalized versus normalized data [9,22], and (ii) correlation [16,21], variance [8,13], or error [18,22] of the normalized *M *values in replicated data.

To ensure that biological variations are retained after normalization, several functional criteria have been employed. Prevailing approaches determine the ability to predict a fixed number of differentially expressed genes in real or simulated data using quantitative measures based on *t*-statistics [4,11,13,21], adjusted *p*-values [11], and false-discovery rates [23]. However, there is uncertainty associated with these measures, and the true number of differentially expressed genes is unknown. Spike-in data have been used to assess normalization approaches for Affymetrix GeneChip data [14,24,25]. However, external control samples are not widely used for evaluation of normalization methods for cDNA microarrays.

In this paper, we evaluated normalization methods for cDNA microarray data using the *k*-NN *LOOCV *classification error (of biological samples characterized by the gene expression profiles), an alternative quantitative functional measure that is relatively unambiguous, objective and readily computed. We used *k*-NN classifiers because (i) their sensitivity enables us to discriminate between, and hence evaluate normalization techniques, (ii) they are readily available, (iii) they perform well in practice, and (iv) their non-parametric nature means that assumptions about how the data are distributed have little influence on classification performance. Since the primary aim of our evaluation of normalization methods was to assist practitioners in choosing effective data pre-processing schemes, we did not consider factors that may influence classification performance, such as feature selection and distance metrics. We investigated a wide spectrum of well-known and widely available normalization techniques: ten location normalization methods that adjust spatial effect and/or intensity effect (Table 1), and three scale methods that adjust scale differences (Table 3). We applied these methods, individually and in combination (41 strategies in all, Tables 1, 2, 3), to five diverse, published, cancer biology-related cDNA microarray data sets (Table 4), and we generated data sets with spatial effect, intensity effect and scale differences removed to varying degrees. Computing the *LOOCV *classification error of *k*-NNs estimated from these multi- and two-class data sets allowed us to investigate which and how much of the dye effect are removed by the 41 strategies.

**Table 1:** Single-bias-removal location normalization techniques used in this study. These strategies remove spatial- or intensity effect in a single step. The abbreviations are as follows, (for a given microarray), *M*_*l*_: location-normalized log ratio; *median*(*M*): median value of non-normalized log ratios; *lowess*(*rloc*_*i*_, *cloc*_*i*_): **lowess **curve fitted as a function of the row location (*rloc*_*i*_) and column location (*cloc*_*i*_) of spots in PT group *i*; *median*(*M*_*w*_): median value of non-normalized log ratios within the window size determined by *w*; *lowess*(*A*): **lowess **curve fitted to an MA plot of spots on a microarray; *lowess*(*A*_*i*_): **lowess **curve fitted to an MA plot of spots in PT group *i*; *spline*(*A*_*iset*_): spline curve fitted to an MA plot of spots in the invariant set, *iset*; *R*_*l*_: location-normalized *R *value; *qspline*(*G*_*i*_): **qspline **smoothing using geometric mean of the *G *channels of all arrays as a target array; *G*_*l*_: location-normalized *G *value; *qspline*(*R*_*t*_): **qspline **smoothing using geometric mean of the *R *channels of all arrays as a target array.

Name *	Description: Effect/Level	Bioconductor R package/function(parameters)
**N**ONRM	No normalization *M*_*l *_= *M*	marray/maNorm(norm="none")
GMEDIAN	Global *M*_*l *_= *M *- *median*(*M*)	marray/maNorm (norm="median", subset = T)
S**L**LOESS	Spatial/local **lowess ***M*_*l *_= *M *- *loess*(*rloc*_*i*_, *cloc*_*i*_)	marray/maNormMain (f.loc = list(maNorm2D(g="maPrintTip", subset = T, span = 0.4))
S**L**FILTER**W3**	Spatial/Local median filter *M*_*l *_= *M *- *median*(*M*_*w*_), W = 3 × 3	tRMA/SpatiallyNormalise** (M, width = 3, height = 3)
S**L**FILTER**W7**	Spatial/Local median filter *M*_*l *_= *M *- *median*(*M*_*w*_), W = 7 × 7	tRMA/SpatiallyNormalise** (M, width = 7, height = 7)
I**G**LOESS	Intensity/Global **lowess ***M*_*l *_= *M *- *loess*(*A*)	marray/maNorm (norm="loess", subset = TRUE, span = 0.4)
I**L**LOESS	Intensity/Local **lowess ***M*_*l *_= *M *- *loess*(*A*_*i*_)	marray/maNorm (norm="printTipLoess", subset = T, span = 0.4)
I**ST**SPLINE	Intensity/Global spline *M*_*l *_= *M *- *spline*(*A*_*iset*_)	affy/normalize.invariantset**(prd.td = c(0.003, 0.007))
QSPLINEG	Intensity/Global **qspline ** *R*_*l *_= *R *- *qspline*(*G*_*t*_), *G*_*l *_= *G *- *qspline*(*G*_*t*_), *M*_*l *_= log(*R*_*l *_/ *G*_*l*_)	affy/*R*_*l *_← normalize.qspline(R, 2^rowMeans(log2(G), na.rm = T), na.rm = T, *default*) *G*_*l *_← normalize.qspline(G, 2^rowMeans(log2(G), na.rm = T), na.rm = T, *default*)
Q**S**PLINER	Intensity/Global **qspline ** *R*_*l *_= *R *- *qspline*(*R*_*t*_), *G*_*l *_= *G *- *qspline*(*R*_*t*_), *M*_*l *_= log(*R*_*l *_/ *G*_*l*_)	affy/ *R*_*l *_← normalize.qspline(R, 2^rowMeans(log2(R), na.rm = T), na.rm = T, *default*) *G*_*l *_← normalize.qspline(G, 2^rowMeans(log2(R), na.rm = T), na.rm = T, *default*)

* We adopted the terminology given in the table to avoid confusion within this work. Elsewhere, these methods are known as: GMEDIAN, global or median [4]; S**L**LOESS, 2D spatial [12]; S**L**FILTER**W3**, spatial normalization using median filter of the block size 3 × 3 [17]; S**L**FILTER**W7**, spatial normalization using median filter of the block size 7 × 7 [17]; I**G**LOESS, global loess [4, 26]; I**L**LOESS, print-tip loess [4]; I**ST**SPLINE, invariant set normalization [38]; Q**S**PLINER, **qspline **using geometric mean of the *R *channels of all arrays as the target array [13]; **Q**SPLINE**G**, **qspline **using geometric mean of the *G *channels of all arrays as the target array [13].

** The SpatiallyNormalise function in the tRMA package was modified to remove scale normalization. The normalize.invariantset function in Affy package was modified so that the function could be applied on cDNA microarray data.

*default* The default parameters for **Q**SPLINE**G** and Q**S**PLINER are (fit.iters = 5, min.offset = 5, spline.method="natural", smooth = T, spar = 0, p.min = 0, p.max = 1.0, incl.ends = T, converge = F)

**Table 2:** Double-bias-removal location normalization techniques used in this study. These strategies remove both spatial- and intensity effect either in a single step (I**G**S**G**LOESS) or in two steps (the remaining thirteen approaches) by combining methods listed in Table 1.

Name	Description: Method/Effect/Level
I**G**S**G**LOESS*	Joint Intensity/Global & Spatial/Global *M*_*l *_= *M *- *lowess*(*A*, *rloc*, *cloc*)

I**G**LOESS-S**L**LOESS	Step 1: I**G**LOESS/Intensity/Global **lowess** Step 2: S**L**LOESS/Spatial/Local **lowess**

I**L**LOESS-S**L**LOESS	Step 1: I**L**LOESS/Intensity/Local **lowess** Step 2: S**L**LOESS/Spatial/Local **lowess**

I**G**LOESS-S**L**FILTER**W3**	Step 1: I**G**LOESS/Intensity/Global **lowess** Step 2: S**L**FILTER**W3**/Spatial/Local median filter

I**G**LOESS-S**L**FILTER**W7**	Step 1: I**G**LOESS/Intensity/Global **lowess** Step 2: S**L**FILTER**W7**/Spatial/Local median filter

I**ST**SPLINE-S**L**LOESS	Step 1: I**ST**SPLINE/Intensity/Global spline Step 2: S**L**LOESS/Spatial/Local **lowess**

I**ST**SPLINE-S**L**FILTER**W3**	Step 1: I**ST**SPLINE/Intensity/ Global spline Step 2: S**L**FILTER**W3**/Spatial/Local median filter

I**ST**SPLINE-S**L**FILTER**W7**	Step 1: I**ST**SPLINE/Intensity/Global spline Step 2: S**L**FILTER**W7**/Spatial/Local median filter

**Q**SPLINE**G**-S**L**LOESS	Step 1: **Q**SPLINE**G**/Intensity/Global **qspline** Step 2: S**L**LOESS/Spatial/Local **lowess**

**Q**SPLINE**G**-S**L**FILTER**W3**	Step 1: **Q**SPLINE**G**/Intensity/Global **qspline** Step 2: S**L**FILTER**W3**/Spatial/Local median filter

**Q**SPLINE**G**-S**L**FILTER**W7**	Step 1: **Q**SPLINE**G**/Intensity/Global **qspline** Step 2: S**L**FILTER**W7**/Spatial/Local median filter

Q**S**PLINER-S**L**LOESS	Step 1: Q**S**PLINER/Intensity/Global **qspline** Step 2: S**L**LOESS/Spatial/Local **lowess**

Q**S**PLINER-S**L**FILTER**W3**	Step 1: Q**S**PLINER/Intensity/Global **qspline** Step 2: S**L**FILTER**W3**/Spatial/Local median filter

Q**S**PLINER-S**L**FILTER**W7**	Step 1: Q**S**PLINER/Intensity/Global **qspline** Step 2: S**L**FILTER**W7**/Spatial/Local median filter

* I**G**S**G**LOESS was implemented in the following package/function: MAANOVA R package/smooth (method="rlowess", f = 0.4, degree = 2). Elsewhere, I**G**S**G**LOESS is known as joint loess [21]. *lowess*(*A*, *rloc*, *cloc*): **lowess **curve fitted as a function of average log intensity (*A*), row location (*rloc*), and column location (*cloc*) of spots on a microarray.

**Table 3:** Extant scale normalization techniques used in this study. For a given microarray, if *M*_*l *_is a location-normalized log ratio, then *M*_*s *_is the scale-normalized log ratio, where *M*_*s *_= *M*_*l *_/ *s*, and *s *is median absolute deviation from the median (*MAD*), a robust estimate of the scale of the data distribution. The remaining abbreviations are as follows, *median*(*M*_*l*_): median value of *M*_*l *_values of spots on all microarrays in a data set; : median value of *M*_*l *_values of spots in PT group *i *on a microarray.

Name *	Description	Bioconductor R package/function (parameters)
**W**SCALE	Within-microarray scale normalization *M*_*s *_= *M*_*l *_/ *s*_*i*_	marrayNorm/maNormScale (norm="printTipMAD", subset = T, geo = T, Mscale = T)

**B**SCALE	Between-microarray scale normalization *s *= *median*(*M*_*l *_- *median*(*M*_*l*_)) *M*_*s *_= *M*_*l *_/ *s*	marrayNorm/maNormScale (norm="globalMAD", subset = T), geo = T, Mscale = T)

**WB**SCALE	Step 1: Within-microarray scale normalization	marrayNorm/maNormScale (norm="printTipMAD", subset = T, geo = T, Mscale = T)
	Step 2: Between-microarray scale normalization *s *= *median*(*M*_*l *_- *median*(*M*_*l*_))	marrayNorm/maNormScale (norm="globalMAD", subset = T, geo = T, Mscale = T)

* We adopted the terminology given in this table to avoid confusion within this work. Elsewhere, the methods are known as: **W**SCALE, within-print-tip-group scale normalization [4]; and **B**SCALE, between slide scale normalization [4, 15].

**Table 4:** The multi-class, cancer-biology related transcriptional profiling data sets analyzed in this work. For each of the five published studies, the fluorescent intensities, microarray images, and associated information were downloaded from the URLs indicated. The statistics refer to data sets produced after application of all pre-normalization data processing, location/scale normalization, and post-normalization data processing steps. The abbreviations are as follows, Microarrays: number of cDNA microarrays; Probes: number of probes; *K*: total number of categories to which a sample could be assigned; Samples and Class: number of samples in the specified pre-defined category; Configuration: configuration of a microarray using the convention described in Figure 1.

Data set name	Description
LIVER CANCER [46]	Microarrays: 181; Probes: 6,605; *K *= 2 Samples and Class: 76 normal; 105 tumor Configuration: (ngr = 8, ngc = 4, nsr = 27, nsc = 28)

LYMPHOMA [47]	Microarrays: 81; Probes: 6,850; *K *= 3 Samples and Class: 29 normal, 43 diffuse large B-cell lymphoma (DLBCL); 9 follicular lymphoma (FL) Configuration: (ngr = 4, ngc = 4, nsr = 24, nsc = 24); (ngr = 8, ngc = 4, nsr = 24, nsc = 24)

RENAL CELL CARCINOMA [48]	Microarrays: 38; Probes: 13,608; *K *= 4 Samples and Class: 3 normal; 26 clear cell carcinoma (CCC); 5 granular cell carcinoma (GCC); 4 papillary carcinoma (PC) Configuration: (ngr = 8, ngc = 4, nsr = 27, nsc = 28)

GASTRIC CARCINOMA [49]	Microarrays: 130; Probes: 15,541; *K *= 2 Samples and Class : 28 normal; 102 tumor Configuration: (ngr = 12, ngc = 4, nsr = 30, nsc = 32); (ngr = 12, ngc = 4, nsr = 29, nsc = 32); (ngr = 12, ngc = 4, nsr = 30, nsc = 30)

LUNG CANCER [50]	Microarrays: 60; Probes: 20,601; *K *= 5 Samples and Class: 6 normal; 35 adenocarcinoma (AC); 11 squamous cell carcinoma (SCC); 4 large cell lung cancer (LCLC); 4 small cell lung cancer (SCLC) Configuration: (ngr = 8, ngc = 4, nsr = 27, nsc = 28)

## Results

### Spatial- and intensity-dependent normalization

#### Diagnostic plots

**Figure 2:**
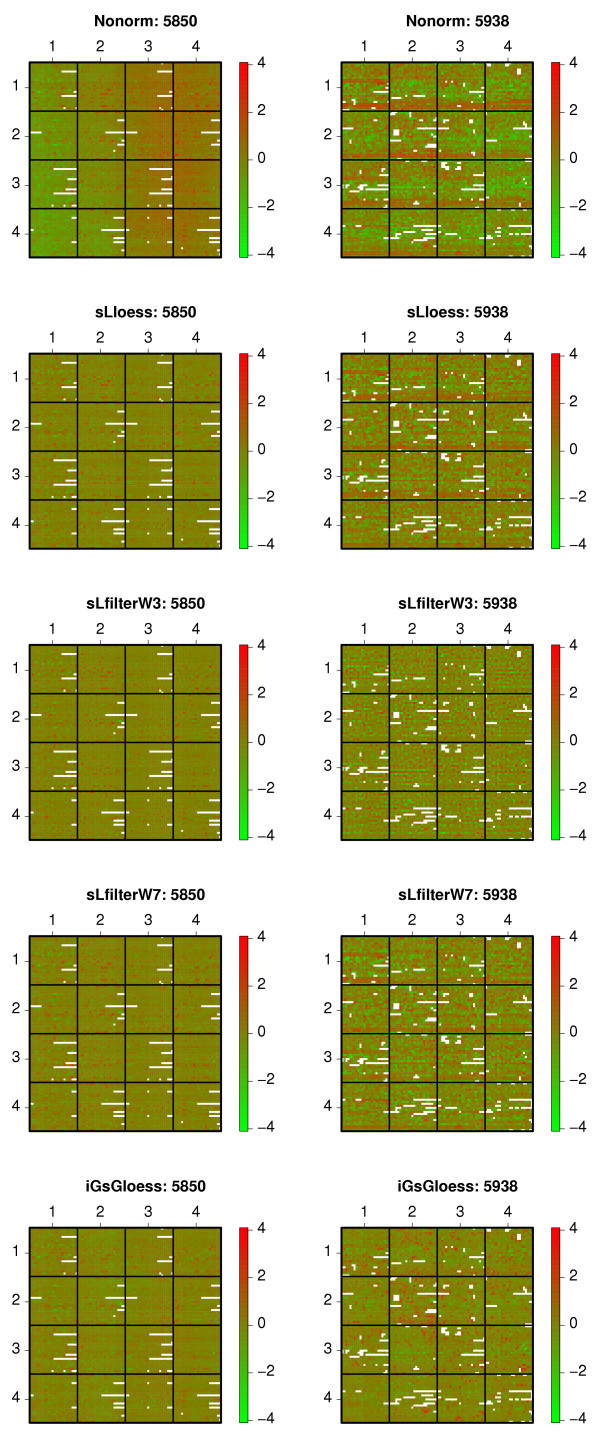
**Spatial plots of microarrays 5850 and 5938 in the Lymphoma data set**. Spatial plots of microarrays 5850 and 5938 in the LYMPHOMA data set. The plots show the results before and after location normalization designed to remove spatial effect. The spatial plot is a spatial representation of spots on the microarray color-coded by their *M *values (**marrayPlots/maImage(x="maM", subset = T)**). Spots in white are spots flagged in the original microarray data (missing values). Rows depict non-normalized (**N**ONRM), and normalized *M*_*l *_values (S**L**LOESS, S**L**FILTER**W3**, S**L**FILTER**W7**, I**G**S**G**LOESS).

We used diagnostic plots to examine the ability of different location normalization methods to remove spatial- and/or intensity effect (Tables 1 and 2). Figure 2 shows spatial plots for two specific LYMPHOMA microarrays normalized with four approaches designed to correct spatial effect (S**L**LOESS, S**L**FILTER**W3**, S**L**FILTER**W7**, I**G**S**G**LOESS). The non-normalized *M *values (**N**ONRM) for microarray "5850" display global spatial effect (left-to-right, green-to-red pattern) whereas those for microarray "5938" exhibit local spatial effect (top-to-bottom, green-to-red pattern in each PT group). Removal of spatial effect should result in a "random" red and green pattern of *M *values. S**L**LOESS and S**L**FILTER**W7** exhibit similar dye bias-removal abilities in that they both remove global spatial effect more effectively than local spatial effect. S**L**FILTER**W3** removes both global and local dye effect effectively, largely because it uses a median filter of a small window size (3 × 3 spots) for normalization. I**G**S**G**LOESS removes most, but not all, global and local spatial effect (a strip of red spots on the right side of "5850" and on the bottom of the PT groups in the first row of "5938" remain). I**G**S**G**LOESS may not be as effective at removing dye effect as expected because, as the developers indicate, **lowess **curve construction uses the standardized spatial variables (*rloc*, *cloc*), which may not be appropriate for location variables [21].

**Figure 3:**
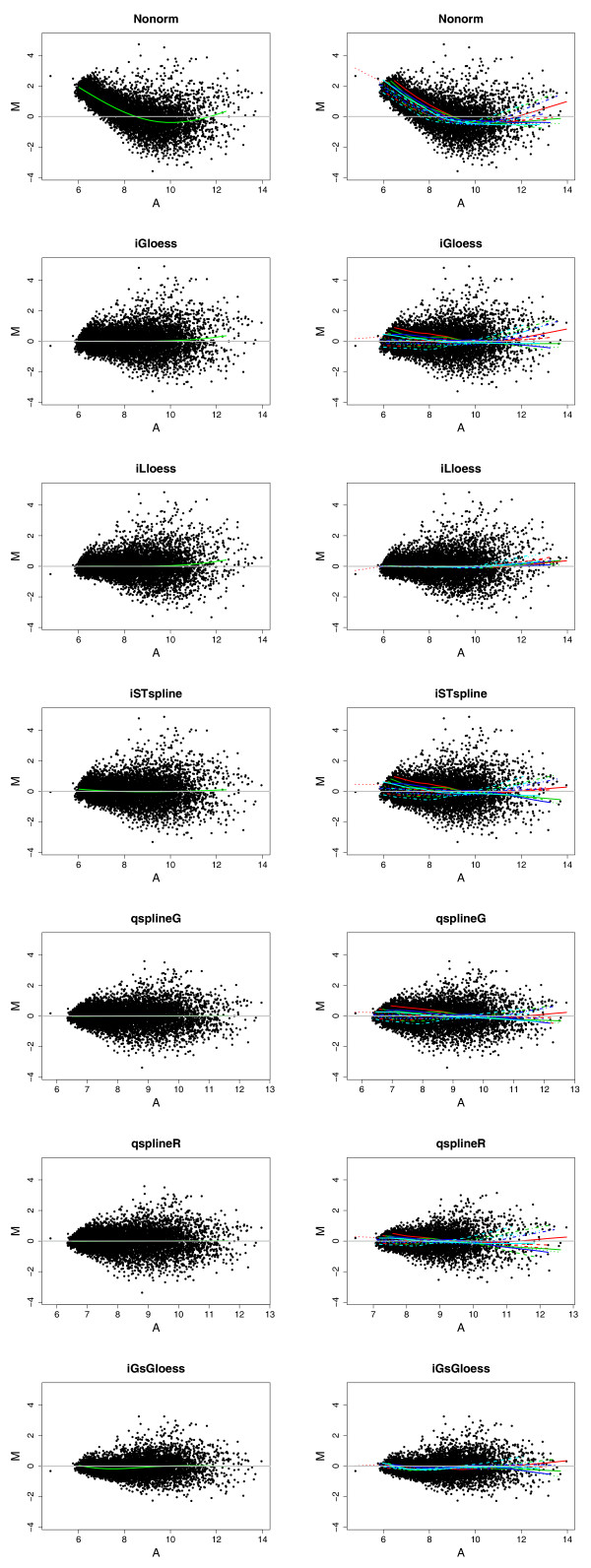
**MA plots of microarray 5812 in the LYMPHOMA data set**. The plots show the results before and after location normalization designed to remove intensity effect. The MA plot is a scatter plot of log ratio *M *= log_2_(*R*_*f *_/ *G*_*f*_) (abscissa) versus average log intensity  (ordinate). Columns depict non-normalized (**N**ONRM), and normalized *M*_*l *_values (I**G**LOESS, I**L**LOESS, I**ST**SPLINE, **Q**SPLINE**G**, Q**S**PLINER, I**G**S**G**LOESS). Plots in the same row represent same data except that each plot in the left panel shows one **lowess **curve for all the spots (**marrayPlots/maPlot(data, z = NULL)**); while that in the right panel shows one **lowess **curve per PT group (**marrayPlots/maPlot(x="maA", y="maM", z="maPrintTip")**). Different colors and line types are used to represent different groups from different rows ("ngr", Figure 1) and columns ("ngc") respectively.

Figure 3 shows intensity-dependent MA plots for one specific LYMPHOMA microarray overlaid with one **lowess **curve (left) or one **lowess **curve per print tip group (right) using six methods designed to correct intensity effect (I**G**LOESS, I**L**LOESS, I**ST**SPLINE, **Q**SPLINE**G**, Q**S**PLINER, I**G**S**G**LOESS). For non-normalized *M *values (**N**ONRM), the curvature in the MA plot indicates the presence of intensity effect at the array (left) and PT (right) level. All six methods remove global intensity effect completely (flat **lowess **curves, left), but only I**L**LOESS and I**G**S**G**LOESS remove local intensity effect thoroughly (right).

Visual inspection of the diagnostic plots in Figures 2 and 3 suggest that S**L**FILTER**W3** is an effective method for removing both global and local spatial effect, whereas I**L**LOESS is good at removing intensity effect.

##### k-NN LOOCV Classification error

**Figure 4:**
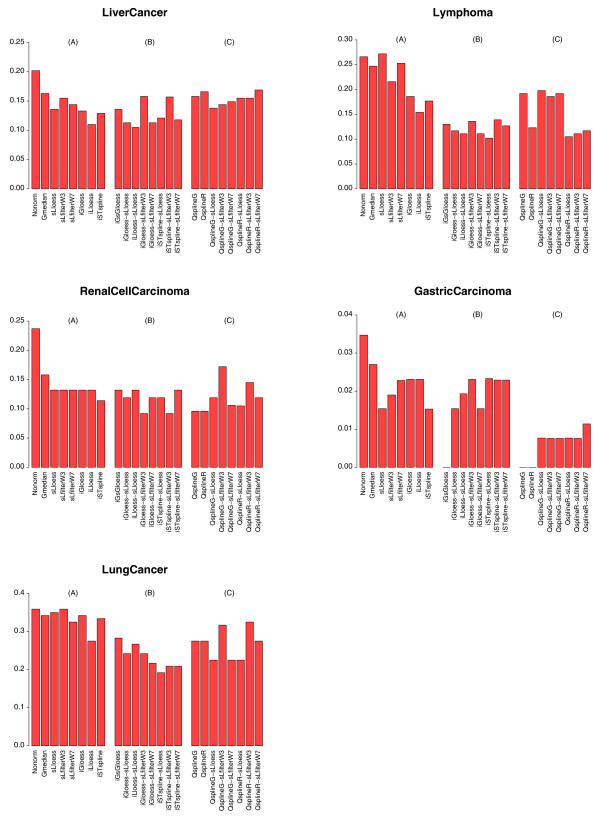
**Bar plots of leave-one-out cross-validation error rates for *k*-NNs in Table 5**. The classifiers were estimated from five data sets (Table 4) either without normalization (**N**ONRM) or normalized using twenty-three normalization techniques that remove spatial- and/or intensity effect to varying degrees (Tables 1 and 2). In each plot, the normalization methods are arranged in the following order: (A) Methods that remove no dye bias (GMEDIAN), or a single dye bias (S**L**LOESS, S**L**FILTER**W3**, S**L**FILTER**W7**, I**G**LOESS, I**L**LOESS, I**ST**SPLINE). (B) Methods that remove two dye biases (I**G**S**G**LOESS, I**G**LOESS-S**L**LOESS, I**L**LOESS-S**L**LOESS, I**G**LOESS-S**L**FILTER**W3**, I**G**LOESS-S**L**FILTER**W7**, I**ST**SPLINE-S**L**LOESS, I**ST**SPLINE-S**L**FILTER**W3**, I**ST**SPLINE-S**L**FILTER**W7**). (C) **Qspline**-related methods (**Q**SPLINE**G**, Q**S**PLINER, **Q**SPLINE**G**-S**L**LOESS, **Q**SPLINE**G**-S**L**FILTER**W3**, **Q**SPLINE**G**-S**L**FILTER**W7**, Q**S**PLINER-S**L**LOESS, Q**S**PLINER-S**L**FILTER**W3**, Q**S**PLINER-S**L**FILTER**W7**).

**Figure 5:**
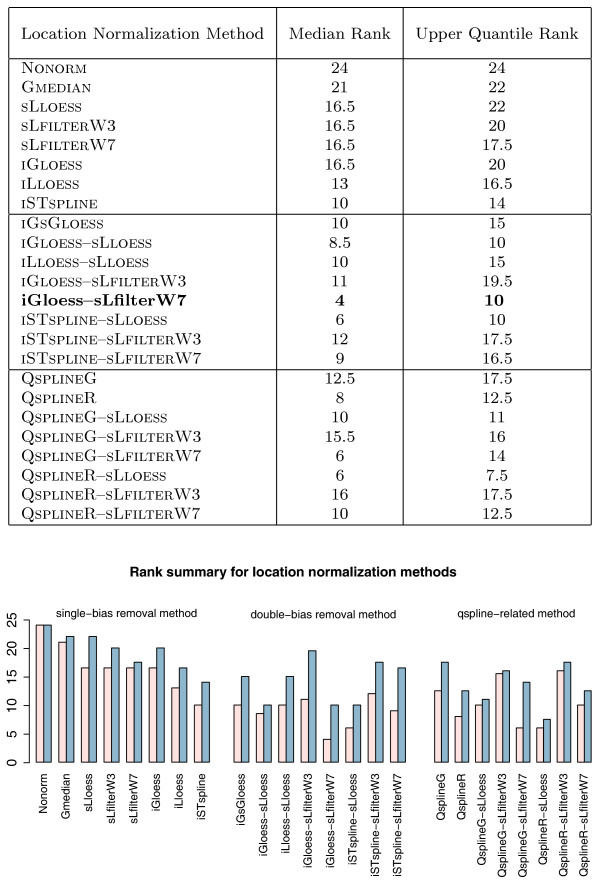
**Rank summary for location normalization methods**. The median and upper quantile ranks of each method are defined as the median and upper quantile values of the ranks of each method across all five data sets (see Table 5, "Ranks"). The bar plots present a visual depiction of the results in the table. (Median ranks are shown in pink; upper quantile ranks are shown in blue.)

For a functional, quantitative evaluation of location normalization methods, we first computed *k*-NN *LOOCV *classification error rates for data normalized using these methods individually and/or in combination. Then for each data set, we ranked the normalization methods based on their *LOOCV *classification error rates. The smaller the *LOOCV *classification error rate, the lower the rank of the normalization strategy. In order to assess whether normalization is beneficial (or not), we also computed the following quantity for a normalization method in each data set:

IMPROVEMENT = (ErrorRate(**N**ONRM) - ErrorRate(Method)) / ErrorRate(**N**ONRM) × 100%

where ErrorRate(**N**ONRM) is the error rate of **N**ONRM, and ErrorRate(Method) is the error rate of the method. Tables 5 and 6 give results for five data sets (Table 4) and 23 location methods designed to remove spatial- and/or intensity effect (Tables 1 and 2). Figures 4 and 5 are alternative, visual representations of the classification "Error Rate" and "Rank" in Table 5.

**Table 5:** Leave-one-out cross-validation *k*-NN error rates for location normalized data. For each data set, the normalization methods were ranked based on their *LOOCV *classification error rates ("Rank"). The smaller the *LOOCV *classification error rate, the lower the rank. The methods are arranged in the following order: single-bias-removal methods (block 1), double-bias-removal methods (block 2) and the **qspline**-related methods (block 3). For a given data set, the smallest error rate(s) and rank(s) are shown in bold. The methods and data sets are described in Tables 1, 2 and 4, respectively.

Location Normalization method	LIVER CANCER		LYMPHOMA		RENAL CELL CARCINOMA		GASTRIC CARCINOMA		LUNG CANCER	
	
	Error Rate	Rank	Error Rate	Rank	Error Rate	Rank	Error Rate	Rank	Error Rate	Rank
**N**ONRM	0.202	24	0.266	23	0.237	24	0.0347	24	0.359	23.5
GMEDIAN	0.163	21	0.247	21	0.158	22	0.0270	23	0.342	20.5
S**L**LOESS	0.136	9.5	0.272	24	0.132	16.5	0.0154	12	0.350	22
S**L**FILTER**W3**	0.155	16	0.216	20	0.132	16.5	0.0190	14	0.359	23.5
S**L**FILTER**W7**	0.144	12.5	0.253	22	0.132	16.5	0.0228	16	0.325	17.5
I**G**LOESS	0.133	8	0.186	15.5	0.132	16.5	0.0231	20	0.342	20.5
I**L**LOESS	0.110	2	0.154	13	0.132	16.5	0.0231	20	0.275	12.5
I**ST**SPLINE	0.129	7	0.177	14	0.114	7	0.0153	10	0.334	19

I**G**S**G**LOESS	0.136	9.5	0.130	10	0.132	16.5	**0**	**2**	0.283	15
I**G**LOESS-S**L**LOESS	0.113	3.5	0.117	6.5	0.119	10	0.0154	12	0.242	8.5
I**L**LOESS-S**L**LOESS	**0.105**	**1**	0.111	4	0.132	16.5	0.0193	15	0.267	10
I**G**LOESS-LFILTERW3	0.158	19.5	0.136	11	**0.092**	**1.5**	0.0231	20	0.242	8.5
I**G**LOESS-S**L**FILTER**W7**	0.113	3.5	0.111	4	0.119	10	0.0154	12	0.217	4
I**ST**SPLINE-S**L**LOESS	0.121	6	**0.102**	**1**	0.119	10	0.0233	22	**0.192**	**1**
I**ST**SPLINE-S**L**FILTER**W3**	0.157	18	0.139	12	**0.092**	**1.5**	0.0229	17.5	0.209	2.5
II**ST**SPLINE-S**L**FILTER**W7**	0.118	5	0.127	9	0.132	16.5	0.0229	17.5	0.209	2.5

**Q**SPLINE**G**	0.158	19.5	0.192	17.5	0.096	3.5	**0**	**2**	0.275	12.5
Q**S**PLINER	0.166	22	0.123	8	0.096	3.5	**0**	**2**	0.275	12.5
**Q**SPLINE**G**-S**L**LOESS	0.138	11	0.198	19	0.119	10	0.00769	7.5	0.225	6
**Q**SPLINE**G**-S**L**FILTER**W3**	0.144	12.5	0.186	15.5	0.172	23	0.00758	5	0.317	16
**Q**SPLINE**G**-S**L**FILTER**W7**	0.149	14	0.192	17.5	0.106	6	0.00758	5	0.225	6
Q**S**PLINER-S**L**LOESS	0.155	16	0.105	2	0.105	5	0.00769	7.5	0.225	6
Q**S**PLINER-S**L**FILTER**W3**	0.155	16	0.111	4	0.145	21	0.00758	5	0.325	17.5
Q**S**PLINER-S**L**FILTER**W7**	0.169	23	0.117	6.5	0.119	10	0.0114	9	0.275	12.5

**Table 6:** IMPROVEMENT of location normalization methods. IMPROVEMENT is defined (in the Results) based on improvement of *LOOCV *classification error rate of a given normalization method over that of **N**ONRM. The methods are arranged in the same order as those in Table 5. For a given data set, the biggest IMPROVEMENT(s) is shown in bold. The methods and data sets are described in Tables 1, 2 and 4, respectively.

Location Normalization method	IMPROVEMENT (%, LIVER CANCER)	IMPROVEMENT (%, LYMPHOMA)	IMPROVEMENT (%, RENAL CELL CARCINOMA)	IMPROVEMENT (%, GASTRIC CARCINOMA)	IMPROVEMENT (%, LUNG CANCER)	IMPROVEMENT RANGE (%)
**N**ONRM	0	0	0	0	0	0 - 0
GMEDIAN	19	7	33	22	5	5 – 33
S**L**LOESS	33	-2	44	56	3	-2 – 56
S**L**FILTER**W3**	23	19	44	45	0	0 – 45
S**L**FILTER**W7**	29	5	44	34	9	5 – 44
I**G**LOESS	34	30	44	33	5	5 – 44
I**L**LOESS	46	42	44	33	23	23 – 46
I**ST**SPLINE	36	33	52	56	7	7 – 56

I**G**S**G**LOESS	33	51	44	**100**	21	21 – 100
I**G**LOESS-S**L**LOESS	44	56	50	56	33	33 – 56
I**L**LOESS-S**L**LOESS	**48**	58	44	44	26	26 – 58
I**G**LOESS-S**L**FILTER**W3**	22	49	**61**	33	33	22 – 61
I**G**LOESS-S**L**FILTER**W7**	44	58	50	56	40	40 – 58
I**ST**SPLINE-S**L**LOESS	40	**62**	50	33	**47**	33 – 62
I**ST**SPLINE-S**L**FILTER**W3**	22	48	**61**	34	42	22 – 61
II**ST**SPLINE-S**L**FILTER**W7**	42	52	44	34	42	34 – 52

**Q**SPLINE**G**	22	28	59	**100**	23	22 – 100
Q**S**PLINER	18	54	59	**100**	23	18 – 100
**Q**SPLINE**G**-S**L**LOESS	32	26	50	78	37	26 – 78
**Q**SPLINE**G**-S**L**FILTER**W3**	29	30	27	78	12	12 – 78
**Q**SPLINE**G**-S**L**FILTER**W7**	26	28	55	78	37	26 – 78
Q**S**PLINER-S**L**LOESS	23	61	56	78	37	23 – 78
Q**S**PLINER-S**L**FILTER**W3**	23	58	39	78	9	9 – 78
Q**S**PLINER-S**L**FILTER**W7**	16	56	50	67	23	16 – 67

###### Single-bias-removal methods

These strategies can be classified into two categories, spatial-dependent and intensity-dependent normalization methods. Three spatial-dependent normalization methods (S**L**LOESS, S**L**FILTER**W3**, S**L**FILTER**W7**) reduce *k*-NN *LOOCV *classification error rates to a similar extent (Tables 5 and 6) and have almost identical ranks (Figure 5), despite the fact that their abilities to remove spatial effect are quite different (Figure 2). Since both S**L**LOESS and S**L**FILTER**W7** fail to remove local spatial patterns effectively (Figure 2, rows 2 and 4), S**L**FILTER**W3** may be too aggressive in removing "dye effect" (Figure 2, row 3). However, the three intensity-dependent methods (I**G**LOESS, I**L**LOESS, I**ST**SPLINE) reduce *k*-NN *LOOCV *classification error rates to different degrees. The *k*-NN *LOOCV *classification error rate and rank of I**G**LOESS are similar to those of the three spatial-dependent methods (S**L**LOESS, S**L**FILTER**W3**, S**L**FILTER**W7**) (Figure 5), whereas I**L**LOESS, which removes intensity effect more completely than I**G**LOESS, has smaller *k*-NN *LOOCV *classification error rates than I**G**LOESS in all five data sets. I**ST**SPLINE, which uses a rank invariant set for normalization, is also better than I**G**LOESS in all five data sets (Figure 5).

In all five data sets, except for LYMPHOMA (S**L**LOESS), the single-bias-removal normalization methods consistently yield smaller *LOOCV *classification error rates than no-bias-removal methods, **N**ONRM and GMEDIAN (which only sets the median of the distribution of *M *values to zero). The greatest benefit, an IMPROVEMENT of 56%, is seen with GASTRIC CARCINOMA (S**L**LOESS, I**ST**SPLINE) (Table 6).

###### Double-bias-removal methods

I**G**S**G**LOESS removes both spatial- and intensity effect in one step, whereas the remaining seven approaches are two-step strategies consisting of single-bias-removal methods applied sequentially (first a method to remove intensity effect, followed by a method to remove spatial effect).

In general, double-bias-removal methods have smaller *k*-NN *LOOCV *classification error rates and bigger IMPROVEMENT than single-bias-removal methods, and all perform better than **N**ONRM and GMEDIAN (Tables 5 and 6, Figures 4 and 5). Using an arbitrary cut-off value of 10 for both median and upper quantile ranks (Figure 5), I**G**LOESS-S**L**FILTER**W7**, I**ST**SPLINE-S**L**LOESS and I**G**LOESS-S**L**LOESS (all of which remove intensity effect globally and then spatial effect locally) appear to be the best methods overall. These three two-step strategies not only have the lowest ranks amongst all normalization methods and across all data sets (Figure 5), they also showed most consistent and significant IMPROVEMENT over both **N**ONRM and GMEDIAN across all five data sets (Table 6). The benefits of using I**G**LOESS-S**L**FILTER**W7** over no normalization (**N**ONRM) range from an IMPROVEMENT value of 40% in LUNG CANCER to 58% in LYMPHOMA (Table 6), whereas the IMPROVEMENT values of I**ST**SPLINE-S**L**LOESS range from 33% in GASTRIC CARCINOMA to 62% in LYMPHOMA and the IMPROVEMENT values of I**G**LOESS-S**L**LOESS range from 33% in LUNG CANCER to 56% in GASTRIC CARCINOMA.

The ranks of the S**L**FILTER**W3**-related approaches (I**G**LOESS-S**L**FILTER**W3**, I**ST**SPLINE-S**L**FILTER**W3**, **Q**SPLINE**G**-S**L**FILTER**W3**, Q**S**PLINER-S**L**FILTER**W3**) are higher than their S**L**FILTER**W7** counterparts (Figure 5), suggesting that a window size of 7 × 7 is more preferable than that of 3 × 3. A smaller window size may over normalize the data, and thus conceal real biological variations.

Compared to the two-step approaches, the rank of the one-step method, I**G**S**G**LOESS, is higher than I**G**LOESS-S**L**FILTER**W7** and I**ST**SPLINE-S**L**LOESS (yet lower than I**G**LOESS-S**L**FILTER**W3** and I**ST**SPLINE-S**L**FILTER**W3**). This indicates that the one-step I**G**S**G**LOESS has no apparent advantage over the two-step bias-removal strategies.

Overall, the classification performances of data normalized using the double-bias-removal methods are better than that of **N**ONRM, and the benefits (IMPROVEMENT) of doing so range from 21% in the case of LUNG CANCER (I**G**S**G**LOESS) to 100% in GASTRIC CARCINOMA (I**G**S**G**LOESS) (Table 6).

###### Qspline-related approaches

Unlike the location normalization methods discussed above, **qspline**-related approaches require a target array. **Q**SPLINE**G** and Q**S**PLINER are single-bias-removal techniques and use *G *and *R *respectively as the target array. The reduction in *k*-NN *LOOCV *classification error rates for these methods is quite significant compared to the other single-bias-removal methods. However, it is noticeable that although **Q**SPLINE**G** and Q**S**PLINER produce similar results in almost all data sets, their results are different in LYMPHOMA (Figures 4 and 5). In addition, when **Q**SPLINE**G** or Q**S**PLINER is combined with one of the three spatial-dependent methods, the rank of the resulting double-bias-removal technique is different from that of its counterpart technique (Figure 5). These results suggest that, similar to other baseline array-based normalization methods [14], the performances of the **qSpline**-related methods may also depend on the choice of the target array.

Overall, the classification performance of data normalized using the **qspline**-related methods is better than **N**ONRM by IMPROVEMENT values of 9% in LUNG CANCER (Q**S**PLINER-S**L**FILTER**W3**) and of 100% in GASTRIC CARCINOMA (**Q**SPLINE**G**, Q**S**PLINER). None of these **qSpline**-related methods, however, outperforms the I**G**LOESS-S**L**FILTER**W7** (Table 6).

#### Scale normalization

**Figure 6:**
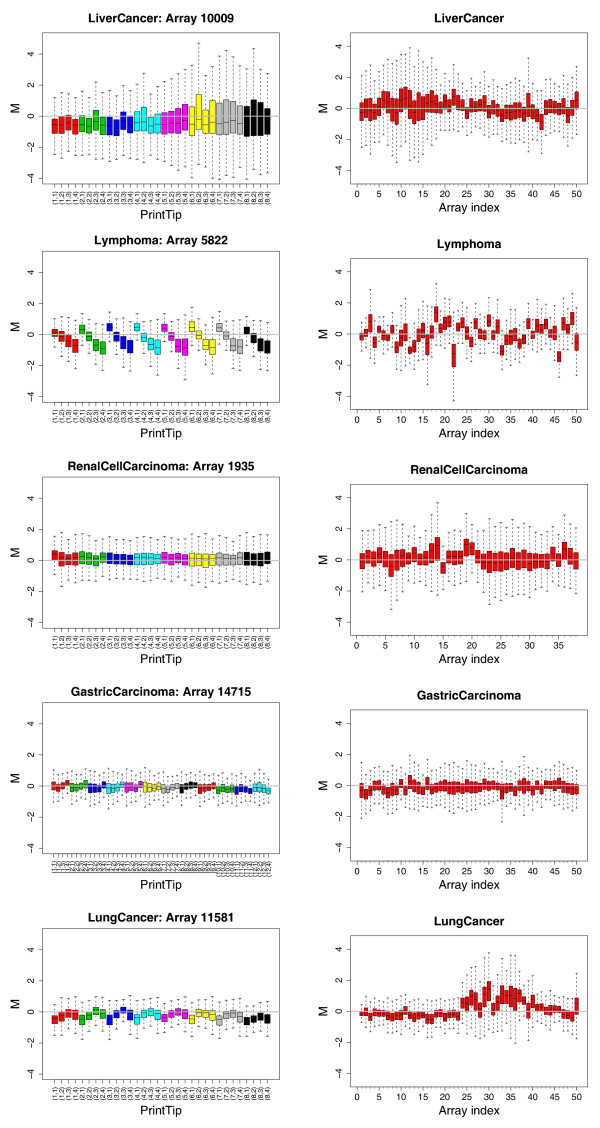
**Boxplots of the distributions of non-normalized *M *values for microarrays in the five studies**. In each boxplot, the box depicts the main body of the data and the whiskers show extreme values. The variability is indicated by the size of the box and the length of the whiskers (**marray/marraymaBoxplot(y="maM")**). Each panel in the left-hand column shows results for *M *values at the local level of a microarray chosen at random from a given data set. The bars are color-coded by PT group. Each panel in the right-hand column shows results for *M *values at the global level for 50 microarrays chosen at random from a given data set (the total number of microarrays in RENAL CELL CARCINOMA is 38). Each row corresponds to a particular study.

Figure 6 shows boxplots of the distribution of non-normalized *M *values for microarrays in the five studies. Scale effect is more apparent between (right) rather than within (left) microarrays in a study. The LYMPHOMA data set shows considerable variations in box size and whisker length both within and between microarrays.

**Figure 7:**
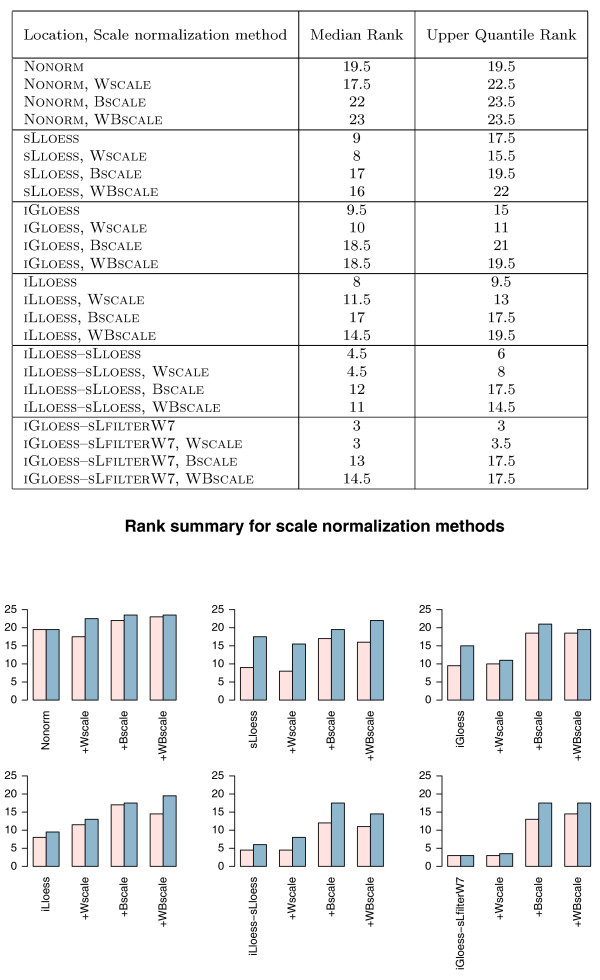
**Rank summary for scale normalization methods**. The median ranks and upper quantile ranks are defined as described in Figure 5. The bar plots present a visual depiction of the results in the table. (Mean ranks are shown in pink; median ranks are shown in blue.) In each plot, normalization strategies are arranged in the following order: a location normalization method, a location normalization method followed by **W**SCALE (+**W**SCALE), a location normalization method followed by **B**SCALE (+**B**SCALE), a location normalization method followed by **WB**SCALE (+**WB**SCALE).

Tables 7 and 8 and Figure 7 show *LOOCV *classification error rates, ranks and IMPROVEMENT for the *k*-NN classifiers estimated using 3 scale normalization methods combined with other spatial- and/or intensity-dependent normalization methods (18 strategies in all). For data normalized first with spatial- and/or intensity-dependent methods, little or no reduction in *LOOCV *classification error rates was observed when within-microarray scale normalization (**W**SCALE) was applied later. However, when between-microarray scale normalization (**B**SCALE) was used alone, or when both scale normalization techniques were used sequentially (**WB**SCALE), there was an increase in both median and upper quantile ranks (Figure 7), suggesting that **B**SCALE should not be applied on the studied data sets. With regard to our running example, the LYMPHOMA data set, scale normalization has no apparent beneficial effect on classification performance.

**Table 7:** Leave-one-out cross-validation k-NN error rates for scale normalized data. Error rate and rank of each scale normalization method. "Rank" is described in detail in Table 5. For a given data set, the smallest error rate(s) and rank(s) are shown in bold. The methods and data sets are described in Tables 3 and 4, respectively.

Location, Scale Normalization method	LIVER CANCER		LYMPHOMA		RENAL CELL CARCINOMA		GASTRIC CARCINOMA		LUNG CANCER	
	
	Error Rate	Rank	Error Rate	Rank	Error Rate	Rank	Error Rate	Rank	Error Rate	Rank
**N**ONRM	0.202	19.5	0.266	18.5	0.237	24	0.0347	14.5	0.359	19.5
**N**ONRM, **W**SCALE	0.185	14.5	0.303	23.5	0.211	22.5	0.0270	12	0.350	17.5
**N**ONRM, **B**SCALE	0.227	23.5	0.272	20.5	0.132	8	0.0615	22	0.425	24
**N**ONRM, **WB**SCALE	0.227	23.5	0.303	23.5	0.132	8	0.0462	21	0.392	23

S**L**LOESS	0.136	9	0.272	20.5	0.132	8	0.0154	3	0.350	17.5
S**L**LOESS, **W**SCALE	0.127	7	0.266	18.5	0.132	8	0.0193	6	0.342	15.5
S**L**LOESS, **B**SCALE	0.202	19.5	0.260	17	0.145	14.5	0.1000	24	0.300	11.5
S**L**LOESS, **WB**SCALE	0.191	16	0.284	22	**0.119**	**2**	0.0846	23	0.283	9.5

I**G**LOESS	0.133	8	0.186	15	0.132	8	0.0231	9.5	0.342	15.5
I**G**LOESS, **W**SCALE	0.141	10	0.148	11	0.132	8	0.0193	6	0.317	13.5
I**G**LOESS, **B**SCALE	0.215	22	0.216	16	0.158	18.5	0.0308	13	0.375	21
I**G**LOESS, **WB**SCALE	0.202	19.5	0.179	14	0.158	18.5	0.0231	9.5	0.383	22

I**L**LOESS	0.110	3.5	0.154	12	0.132	8	0.0231	9.5	0.275	7
I**L**LOESS, **W**SCALE	0.116	6	0.161	13	0.145	14.5	0.0231	9.5	0.300	11.5
I**L**LOESS, **B**SCALE	0.193	17	0.111	3	0.145	14.5	0.0385	17.5	0.359	19.5
I**L**LOESS, **WB**SCALE	0.202	19.5	**0.105**	**1**	0.145	14.5	0.0424	20	0.317	13.5

I**L**LOESS-S**L**LOESS	**0.105**	**1.5**	0.111	3	0.132	8	0.0193	6	0.267	4.5
I**L**LOESS-S**L**LOESS, **W**SCALE	**0.105**	**1.5**	0.136	9.5	0.132	8	**0.00769**	**1**	0.267	4.5
I**L**LOESS-S**L**LOESS, **B**SCALE	0.160	12	0.130	8	0.211	22.5	0.0385	17.5	0.275	7
I**L**LOESS-S**L**LOESS, **WB**SCALE	0.152	11	0.123	6	0.158	18.5	0.0347	14.5	0.259	3

I**G**LOESS-S**L**FILTER**W7**	0.113	5	0.111	3	**0.119**	**2**	0.0154	3	0.217	2
I**G**LOESS-S**L**FILTER**W7**, **W**SCALE	0.110	3.5	0.124	7	**0.119**	**2**	0.0154	3	**0.209**	**1**
I**G**LOESS-S**L**FILTER**W7**, **B**SCALE	0.180	13	0.117	5	0.171	21	0.0385	17.5	0.275	7
I**G**LOESS-S**L**FILTER**W7**, **WB**SCALE	0.185	14.5	0.136	9.5	0.158	18.5	0.0385	17.5	0.283	9.5

**Table 8:** IMPROVEMENT of the scale normalization methods. IMPROVEMENT is described in detail in Table 6. For a given data set, the biggest IMPROVEMENT(s) is shown in bold. The methods and data sets are described in Tables 3 and 4, respectively.

Location, Scale Normalization method	IMPROVEMENT (%, LIVER CANCER)	IMPROVEMENT (%, LYMPHOMA)	IMPROVEMENT (%, RENAL CELL CARCINOMA)	IMPROVEMENT (%, GASTRIC CARCINOMA)	IMPROVEMENT (%, LUNG CANCER)
**N**ONRM	0	0	0	0	0
**N**ONRM, **W**SCALE	8	-13	11	22	3
**N**ONRM, **B**SCALE	-12	-2	44	-77	-18
**N**ONRM, **WB**SCALE	-12	-13	44	-33	-9

S**L**LOESS	33	-2	44	56	3
S**L**LOESS, **W**SCALE	37	0	44	44	5
S**L**LOESS, **B**SCALE	0	2	39	-188	16
S**L**LOESS, **WB**SCALE	5	-7	**50**	-144	21

I**G**LOESS	34	30	44	33	5
I**G**LOESS, **W**SCALE	30	44	44	44	12
I**G**LOESS, **B**SCALE	-6	19	33	11	-4
I**G**LOESS, **WB**SCALE	0	33	33	33	-7

I**L**LOESS	45	42	44	33	23
I**L**LOESS, **W**SCALE	43	39	39	33	16
I**L**LOESS, **B**SCALE	4	58	39	-11	0
I**L**LOESS, **WB**SCALE	0	**61**	39	-22	12

I**L**LOESS-S**L**LOESS	**48**	58	44	44	26
I**L**LOESS-S**L**LOESS, **W**SCALE	**48**	49	44	**78**	26
I**L**LOESS-S**L**LOESS, **B**SCALE	21	51	11	-11	23
I**L**LOESS-S**L**LOESS, **WB**SCALE	25	54	33	0	28

I**G**LOESS-S**L**FILTER**W7**	44	58	**50**	56	40
I**G**LOESS-S**L**FILTER**W7**, **W**SCALE	46	53	**50**	56	**42**
I**G**LOESS-S**L**FILTER**W7**, **B**SCALE	11	56	28	-11	23
I**G**LOESS-S**L**FILTER**W7**, **WB**SCALE	8	49	33	-11	21

## Discussion

This computational investigation employed two types of visual diagnostic plots and *k*-NN *LOOCV *classification error rates to evaluate a broad suite of known normalization strategies. These analyses were applied to cDNA microarray data from five published cancer studies. Since all these data sets were acquired using GenePix image analysis software and a recent study showed that background adjustment using GenePix can increase variability of microarray data and compromise downstream data analyses [3], we used foreground intensity values of the probes without background adjustment in this work. The normalization approaches examined are based on a variety of different techniques and implementations that are readily available and accessible.

Our results show that the *LOOCV *classification error of *k*-NN classifiers depends on how much of spatial- and intensity effect can be removed by a normalization strategy. Overall, the single-bias-removal location approaches perform better than GMEDIAN and **N**ONRM, while the double-bias-removal location strategies perform better than the single-bias-removal location approaches. Of the twenty-three location normalization techniques investigated, three two-step processes (I**G**LOESS-S**L**FILTER**W7**, I**ST**SPLINE-S**L**LOESS and I**G**LOESS-S**L**LOESS), all of which removes intensity effect at the global level and spatial effect at the local level, appear to be the most effective at reducing *LOOCV *classification error. However, removing spatial- or intensity effect alone is not sufficient for reducing *LOOCV *classification error (see below).

A recent review of normalization methods [26] raised the concern that removing spatial effect (S**L**LOESS and the related methods) may add additional noise to normalized data, and suggested that a safe alternative was removing only intensity effect at the local level (I**L**LOESS) [26]. Our results show that, although the classification performance of data normalized with S**L**LOESS alone can be worse than non-normalized data as in the case of the LYMPHOMA data set, when S**L**LOESS is combined with another intensity-dependent approach (I**G**LOESS, I**L**LOESS, I**ST**SPLINE, **Q**SPLINE**G**, or Q**S**PLINER), there is considerable improvement over **N**ONRM, with IMPROVEMENT ranging from 23% in LIVER CANCER (Q**S**PLINER-S**L**LOESS) to 78% in GASTRIC CARCINOMA (Q**S**PLINER-S**L**LOESS, **Q**SPLINE**G**-S**L**LOESS). Thus, removing both spatial- and intensity effect is beneficial for the downstream analytical task of classification. Another study compared various **lowess**-based single-bias-removal intensity normalization approaches, and found that I**L**LOESS may not significantly improve the results compared to I**G**LOESS [27]. Our results show that the benefits (IMPROVEMENT) of I**G**LOESS over **N**ONRM range from 5% in LUNG CANCER to 44% in RENAL CELL CARCINOMA; while that the benefits (IMPROVEMENT) of I**L**LOESS over **N**ONRM range from 23% in RENAL CELL CARCINOMA to 46% in LIVER CANCER. Therefore, I**L**LOESS performs better than I**G**LOESS in our study. However, as a single-bias-removal approach, I**L**LOESS still fail to outperform I**G**LOESS-S**L**FILTER**W7**, I**ST**SPLINE-S**L**LOESS and I**G**LOESS-S**L**LOESS, which are the best overall methods and whose IMPROVEMENT values over **N**ONRM range from 40% in LUNG CANCER to 58% in LYMPHOMA for I**G**LOESS-S**L**FILTER**W7**, from 33% in GASTRIC CARCINOMA to 62% in LYMPHOMA for I**ST**SPLINE-S**L**LOESS and from 33% in LUNG CANCER to 56% in GASTRIC CARCINOMA for I**G**LOESS-S**L**LOESS (Table 6).

A previous study employed *k*-NN classification of diluted samples to assess a small number of global linear methods for normalization [28]. The study presented here is more comprehensive, both in terms of the range of data sets and the diversity of normalization techniques. Our results indicate that the *k*-NN *LOOCV *classification error of real biological samples provides an informative functional quantitative measure that can be used to evaluate normalization approaches.

Differences in scale between microarrays can arise from both unwanted technical factors (differences in experimental reagents, equipment, personnel, and so on), as well as from genuine biological variations. The scale normalization techniques applied here aim to remove unwanted technical factors, and assume the existence of little biological variations between samples. For the five studied data sets, scale normalization of non- or location-normalized data do not result in an overall reduction in *LOOCV *classification error. Indeed, two between-microarray normalization methods (**B**SCALE, **WB**SCALE) result in an overall increase in *LOOCV *classification error (poorer performance, Figure 7). These results suggest that in the examined cancer-related data sets, there can be considerable genuine biological variations (which is plausible because genomic aberrations found in cancer cells [29,30] may alter the number and nature of expressed genes compared to normal cells), and that these variations are masked by the applied scale normalization. The data sets considered here do not contain replicated data, so it is difficult to ascertain how much of the scale effect result from unwanted technical factors. Scale normalization may be warranted in situations where technical differences can be discerned by examination of the replicated data and genuine biological variations are known or believed to exist. In such cases, scale normalization using external control samples may be more useful than the total gene approaches.

While our empirical analyses are thoroughgoing in terms of both normalization procedures and test data sets, we acknowledge that there are two caveats in this study that deserve attention and further investigation. First, we employed the *LOOCV *classification error as a functional measure to assess normalization methods. In principle, *LOOCV *provides an almost unbiased estimate of the generalization ability of a classifier [31], especially when the number of the available training samples is severely limited (as in the case of LYMPHOMA and RENAL CELL CARCINOMA in Table 4), and is thus highly desirable for model selection or other relevant algorithm evaluation [32,33]. However, it is also known that the *LOOCV *error estimator may have high variance in some situations [34,35], which could in turn affect the accuracy of the rankings of the normalization methods. Empirically, however, we found that the *LOOCV *errors we obtained from various round of classification are quite stable, therefore we believe that our estimation is in practice reliable and suitable for ranking. Nevertheless, error estimators that have shown to have low variance (e.g., bootstrapping and *k*-fold cross-validation [34,35]) are worth further investigation in the future.

The second caveat of this work is that normalization methods were evaluated using *k*-NN classification without the aid of auxiliary techniques, such as feature selection. The reasons we did not employ feature selection, but rather used all the probes that are present in the majority of the microarrays for classification are as follow: i) We believe that the influence of the dye effect (which usually affect a large number of the probes) on the downstream data analysis can be better and more consistently reflected when a large number of the probes are examined. As such, using all valid probes for training a classifier can best reflect the effectiveness of the normalization methods, whereas using subsets of the probes may generate inconsistent results due to the heterogeneous nature of the dye effect across microarrays. ii) We also included low intensity probes in the analyses. Although this may add noise and therefore could compromise the absolute classification performance of the examined normalization methods, we nevertheless think that these probes should not be excluded because reducing variability in low intensity probes is by itself an important objective of normalization methods. That is, a good normalization approach should be able to reduce variability in both low intensity- and high intensity probes effectively. And iii) we are aware that *k*-NNs without feature selection may add variability to the classification results, however, *k*-NN classification is also appealing in that it is simple and requires no data pre-processing or assumption on data distribution. In addition, *k*-NN classifiers have been widely used in many classification tasks including high-dimensional problems arising from image and text data [36].

Due to the above two caveats, the relative rankings of the investigated normalization strategies can hardly be obtained accurately in this work. For example, our results show that I**G**LOESS-S**L**FILTER**W7**, I**ST**SPLINE-S**L**LOESS and I**G**LOESS-S**L**LOESS reduce *LOOCV *classification errors most consistently and effectively across all five data sets. It is difficult, however, to determine further which of these three strategies is the best, because small differences in classification results can either arise from inherent differences in these approaches and/or from the variability introduced by the *LOOCV *error estimator and less optimal *k*-NN classifiers. Moreover, our results should not be taken as a warrant of directly using baseline methods, such as *k*-NNs without feature selection, for high-dimensional classification tasks. More investigations are needed to understand the interplay between normalization (which improves data quality) and feature selection (which improves the classifier by throwing away non-informative data) to ascertain normalization strategies to produce an optimal classifier.

## Conclusion

Using *LOOCV *error of *k*-NNs as the evaluation criterion, we assessed a variety of normalization methods that remove spatial effect, intensity effect and scale differences from cDNA microarray data. Overall, the single-bias-removal location approaches (which remove either spatial- or intensity effect from the data) perform better than GMEDIAN and **N**ONRM, while the double-bias-removal location strategies (which remove both spatial- and intensity effect) perform better than the single-bias-removal location approaches. Of the 41 different strategies examined, I**G**LOESS-S**L**FILTER**W7**, I**ST**SPLINE-S**L**LOESS and I**G**LOESS-S**LB**SCALE, all of which are two-step approaches and remove both intensity effect at the global level and spatial effect at the local level, appear to be the most effective at reducing *LOOCV *classification error. The investigated scale normalization methods do not have beneficial effect on classification performance. These results also indicate that spatial- and intensity effect do have profound impact on downstream data analyses, such as classification, and that removing these effects can improve the quality of such analyses.

## Methods

### Extant data sets and software

Table 4 summarizes relevant information on the cDNA microarray data sets from the Stanford Microarray Database (SMD) reexamined here. These data sets were selected because the published studies assayed samples from distinct cancers, the profiling experiments were performed at different times, four out of the five data sets were produced by different investigators, and the microarrays used were printed with different probes on different occasions. The LYMPHOMA study has been used as the illustrative, running example.

A variety of computational tools for manipulating, analyzing, and visualizing microarray data are available. These include open source implementations based on the R language for statistical computing [37] such as the Bioconductor , MAANOVA , tRMA , and braju  packages. Standard R and Bioconductor packages and functions were used apart from one normalization method found in MAANOVA (joint removal of both spatial- and intensity effect at the global microarray level, I**G**SG**B**SCALE) and two normalization methods found in tRMA (removal of spatial effect at the local PT level, S**L**FILTER**W3** and S**L**FILTER**W7**).

### Pre-normalization data processing

For each spot, the foreground red (*R*_*f*_) and green (*G*_*f*_) quantitated fluorescent intensities (acquired using GenePix image analysis software) of the arrayed DNA sequences were used to compute the non-normalized log ratio, *M *= log_2_(*R*_*f *_/ *G*_*f*_), and average log intensity, . Because of the concern that local background values estimated by GenePix may add additional noise to the data [3], these values were not subtracted from their corresponding foreground values. For a given microarray, the log ratios were normalized using location- and/or scale-normalization techniques and its particular configuration (the LYMPHOMA and GASTRIC CARCINOMA studies employed microarrays with two and three distinct configurations respectively).

### Normalization methods

Tables 1 and 2 summarize the 23 location normalization methods that remove none, one, or both of spatial- and intensity effect (detailed descriptions of how they adjust *M *values can be found elsewhere [4,6,12,13,17,21,38,39]). In particular, Table 1 includes two methods that remove no spatial- or intensity-dependent dye bias: (i) **N**ONRM neither removes any effect nor alters the distribution of *M *values; and (ii) GMEDIAN does not remove any effect but acts as a baseline normalization method because it sets the mean or median of *M *to zero. There are eight methods that remove either spatial- or intensity effect: (i) S**LB**SCALE removes spatial effect at the PT level using **lowess**; (ii) S**L**FILTER**W3** and S**L**FILTER**W7** remove spatial effect using median filters of the block sizes 3 × 3 and 7 × 7, respectively [17]; (iii) I**GB**SCALE removes intensity effect at the global level; (iv) I**LB**SCALE removes intensity effect at the local level and as a byproduct removes spatial effect partially; (v) I**ST**SPLINE removes intensity effect at the global level using rank invariant set and a spline smoothing technique [38]; and (vi) Q**S**PLINER (**Q**SPLINE**G**) removes intensity effect at the global level using spline smoothing applied to quantiles obtained from *R *(*G*) and using the geometric mean of the *R *(*G*) channels of all arrays as the target array [13]. In Table 2, I**G**S**G**LOESS is a one-step process that removes global intensity effect and global spatial effect, while the remaining thirteen strategies are two-step processes that remove both dye effect by combining methods in Table 1.

Table 3 summarizes the three scale normalization methods used (detailed descriptions of how these methods adjust the scale of *M *values can be found elsewhere [4]). **W**SCALE adjusts the scale of *M *values at the PT level. **B**SCALE adjusts the scale of *M *values globally across all microarrays in a data set. **WB**SCALE adjusts the scale locally followed by globally, in two steps. These scale normalization methods were applied to non-normalized data (**N**ONRM) and to data that had been normalized using the five location methods S**L**LOESS, I**G**LOESS, I**L**LOESS, I**L**LOESS-S**L**LOESS, or I**G**LOESS-S**L**FILTER**W7**. These methods were selected to represent methods that remove spatial and/or intensity effect at different levels.

### Post-normalization data processing

For the five cancer-biology studies, examination of the published data indicated that probes printed on different microarrays (even those with the same configuration) were not necessarily identical. For the *N *microarrays associated with a given study (*N *can be equated with the value given for "Microarrays" in Table 4), the 41 data sets used to estimate *k*-NN classifiers and to determine their *LOOCV *classification errors were created as follows. Each microarray was handled as described in "Pre-normalization data processing" and the ensuing *M *values were normalized using the 41 distinct location and/or scale techniques discussed above. A probe was retained for further processing only if it was printed and present (*i.e.*, successfully measured and computed) in 95% of the *N *microarrays. If a probe met these criteria, missing *M *values were imputed using the *k*-NNimpute algorithm [40] as implemented in the Bioconductor package/function **pamr/pamr.knnimpute(k = 10) **[41]. Given the 41 data sets, the *M *values for a probe in all *N *microarrays were centered and rescaled to a unit norm. For LYMPHOMA, the final dimensionality (number of probes after post-normalization data processing) of each of the *N *= 81 data points was 6,850 ("Probes"). The 41 post-normalized data sets for the five examined studies are available at .

### Classification error

Given *D *data points, each of which is assigned to one of *K *categories (*e.g.*, "normal", "DLBCL", "FL"), a *LOOCV *procedure for this *K*-class data set is as follows. The data set is partitioned into a test set of one data point and a learning set of *D*-1 data points. The learning set is used to train a classifier and the ensuing model is employed to predict the class label of the test data point. This process is repeated so that the class of each data point is predicted using a classifier estimated from all other data points in the data set. Classification error is the number of the instances in which the predicted class of a data point differs from its known class. The error rate is this value divided by the number of data points, *D*.

#### k-NN classifier

Given a *K*-class data set, the *k*-NN algorithm predicts the class label of a test data point by first finding which of the data points in the data set are its *k *closest neighbors. The classes of these *k *nearest neighbors are examined and the class of the test data point decided by a majority vote, with ties being broken at random. If there are ties for the *k*th nearest data point, all candidates are included in the vote. Classification using *k*-NNs does not require any special handling of multi-class data sets. A widely employed measure of the proximity of two data points and the one utilized here is the standardized Euclidean distance [42,43]. Since all probes are treated with equal weight, the classification results are affected by all the probes rather than just a subset, as would have been the case if feature selection had been employed. Euclidean distance has been shown to be effective and accurate on a variety of data sets [43,44].

The optimal number of nearest neighbors, k*, was determined via leave-one-out cross-validation. An original data set of *D *data points was partitioned into a test set of one data point and a learning set of *D *- 1 data points. Given a specific *k*, the *k*-NN algorithm was used to predict the class of each data point in the learning set using the *D *- 2 remaining data points. The classification error, *ε*_*k*_, of the learning set was determined. This procedure was performed using *k *= {3,...,10} and k* taken to be the *k *producing the smallest classification error, *i.e.*, *min*_*k*_(*ε*_*k*_). The class of the test data point for the original data set was predicted using k* and the *k*-NN algorithm. This entire process was repeated such that each of the data points in the original data set was used as a test set. The classification error of the original data was calculated. The *k*-NN step was performed using the R class/package [45]**class/knn.cv(k = "number of neighbors") **where "number of neighbors" was set to 3,...,10. The prediction step was performed using **class/knn(k = "optimal k") **where "optimal k" was k*.

## List of abbreviations

AC: adenocarcinoma;

CCC: clear cell carcinoma;

DLBCL: diffuse large B-cell lymphoma;

FL: follicular lymphoma;

GCC: granular cell carcinoma;

*k*-NN: *k*-nearest neighbor;

LCLC: large cell lung cancer;

*LOOCV*: Leave-one-out cross-validation;

**lowess**: local regression estimation;

PC: papillary carcinoma;

PT: print-tip group;

SCC: squamous cell carcinoma;

SCLC: small cell lung cancer;

SMD: Stanford Microarray Database.

## Authors' contributions

WW designed and performed computational experiments, and drafted the manuscript. EPX participated in experimental design and in drafting the manuscript. ISM participated in experimental design and edited the manuscript. CM and MJB read and edited the manuscript. All authors contributed to, read and approved the final manuscript.
